# Primary healthcare provider-perceived barriers to implementing an evidence-based pathway for undifferentiated lower gastrointestinal tract symptoms: A qualitative inquiry

**DOI:** 10.1371/journal.pone.0313201

**Published:** 2024-12-31

**Authors:** Sowmya Sharma, Michael J. Stewart, Holly Mathias, Kerri Novak, Sander Veldhuyzen Van Zanten, Courtney Heisler, Sharon Richard, Emily Neil, Frederick Burge, Aaron Smith, Kevork Peltekian, Sunil Patel, Jennifer L. Jones

**Affiliations:** 1 Department of Gastroenterology and Hepatology, John Hopkins University, Baltimore, Maryland, United States of America; 2 Department of Digestive Care and Endoscopy, Dalhousie University, Halifax, Nova Scotia, Canada; 3 School of Public Health, University of Alberta, Edmonton, Alberta, Canada; 4 Division of Gastroenterology and Hepatology, University of Calgary, Calgary, Alberta, Canada; 5 Division of Gastroenterology, University of Alberta, Edmonton, Alberta, Canada; 6 Nova Scotia Health Authority, Halifax, Nova Scotia, Canada; 7 Department of Family Medicine, Dalhousie University, Halifax, Nova Scotia, Canada; University of Kentucky, UNITED STATES OF AMERICA

## Abstract

**Background:**

Primary healthcare providers play a critical role in diagnosing and managing digestive disorders. Standardized clinical care guidelines have been developed, but with limited and inconsistent implementation. An evidence-based gastroenterology clinical care pathway (GUTLINK) has been proposed in one region of Canada; however, little is known in the medical literature about potential barriers to pathway implementation within primary care. We aimed to identify behavioral and environmental barriers and facilitators to implementation of evidence-based care pathways for undifferentiated lower gastrointestinal tract symptoms in primary care.

**Methods:**

One-on-one semi-structured interviews were conducted with primary healthcare providers between September 2021 and May 2022. Interview script development was guided by the COM-B framework. Interviews were transcribed and data were analyzed using an inductive thematic analysis approach.

**Results:**

A total of 15 primary healthcare provider interviews were conducted. Several key barriers to GUTLINK implementation were identified in all three domains of the COM-B framework. Key barriers included Capability (e.g., Physician Knowledge and Access to Allied Health), Opportunity (e.g., Access to diagnostic tools), and Motivation (e.g., Comfort with managing cases and optimism). Some of these barriers have not previously been identified in medical literature.

**Conclusions:**

Evidence-based clinical care pathways have the potential to support access to quality gastroenterology care, yet primary healthcare providers in this study identified several barriers to implementation. Potential solutions exist at the individual and clinic levels (e.g., greater education, improved provider-specialist communication), but must be supported with systems-level changes (e.g., increased funding for gastrointestinal care and e-Health platforms) to support pathway implementation and improve quality of care.

## Introduction

Digestive diseases, such as irritable bowel syndrome (IBS) and inflammatory bowel disease (IBD), are prevalent gastrointestinal (GI) conditions in Canada. It is estimated that over 20 million Canadians suffer from digestive disorders every year, and prevalence has doubled in the last decade (For example, 400 IBD cases per 100,000 in 2002 to 825 IBD cases per 100,000 in 2023) [1–3]. In 2002, the prevalence of IBD in Canada 400 cases per 100,000 people. The prevalence of digestive disease has vast health, social and economic impacts. It is estimated that at least 10% of hospitalizations in Canada are linked to digestive disease [2]. Approximately 30,000 Canadians die from digestive disease annually [2]. Such disease burden has significant direct and indirect costs to the health care system [2–7] with direct healthcare costs for IBD estimated to exceed $28 billion by 2025 [5].

Primary Healthcare Providers (PHCPs) play an important role in the management of digestive disorders with GI conditions comprising up to 20% of cases in primary care practice [1, 8]. PHCPs are often the first point of medical care and are often required to diagnose and provide medical management of patients with digestive disorders. However, patient and PHCP dissatisfaction with access to GI speciality care has been well documented [9, 10].

This research is set in Nova Scotia (NS) Canada, a small province on the Atlantic coast with a population of approximately 1 million people [11]. Despite the relatively small population, NS has the highest population-based prevalence rates of digestive diseases in Canada [4, 7] and population-based need outstrips the capacity of available healthcare resources. The province’s only tertiary care centre in gastroenterology faces wait times well in excess of recommended benchmarks for GI specialty care [1, 8, 9, 12]. Limited resources, variability in referral quality, provincial infrastructure, triage criteria, complexity of health system navigation, and lack of familiarity with evidence-based guidelines in practice may contribute to limited access to GI specialty care. Standardized implementation of evidence-based clinical care pathways has been shown to have the potential to reduce GI specialty referrals (45–80%), reduce colonoscopies (60%), and result in fewer unnecessary tests. Improving uptake of clinical care pathways could lead to improved access to care, enhanced patient quality of life, and less health system waste.

Despite the availability of evidence-based GI clinical care guidelines, implementation is limited and inconsistent in primary care [13, 14]. The application of basic diagnostic criteria for IBS has been observed in 30% of family practices [4, 15]. Subsequent implementation of a GI luminal pathway revealed that more than 70% of patients did not require a referral or colonoscopy [16, 17]. A lack of implementation of evidence-based care guidelines in primary care results in variability in the number and quality of GI referrals, longer wait times, inappropriate testing and treatments, and poor patient quality of life. Implementation of a high-quality, evidence-based clinical care pathway in NS, called GUTLINK, has been proposed. A clinical care pathway is a tool used to “translate clinical practice guideline recommendations into clinical processes of care within the unique culture and environment of a healthcare institution” [18]. GUTLINK is a multifaceted virtual medical neighbourhood that will serve as a virtual interface between healthcare providers. GUTLINK will provide a digital infrastructure that aligns PHCPs, GI specialists and other allied healthcare professionals into a tightly coordinated team to provide local and remote collaborative evidence-based medical care. A first step to pathway implementation includes understanding the barriers to end user pathway use as well as the solutions to these barriers.

The objectives of this research project were to identify behavioral and environmental barriers and facilitators to the successful implementation of evidence-based care pathways for undifferentiated lower GI tract symptoms in primary care.

### Theoretical framework

We used the COM-B framework to guide interview script development and organize the reporting of findings [19]. The implementation of evidence-based practice in healthcare requires behaviour change. The COM-B framework was developed through a systematic review of existing behaviour change interventions to create a behaviour change wheel for implementation science. It proposes that behaviour change can occur when addressing three key aspects of the behaviour system: capability, opportunity, and motivation. Capability is defined as “the individual’s psychological and physical capacity to engage in the activity concerned”. Opportunity comprises “all the factors that lie outside the individual that make the behaviour possible or prompt it”, and Motivation includes “brain processes that energize and direct behaviour, not just goals and conscious decision-making” [19].

## Methods

### Aim

To identify behavioral and environmental barriers and facilitators to the successful implementation of evidence-based care pathways for undifferentiated lower GI tract symptoms in primary care.

### Setting

Data collection took place in Nova Scotia–a small province of approximately 1 million people situated on the Atlantic coast of Canada.

### Study design

**Fig 1:**
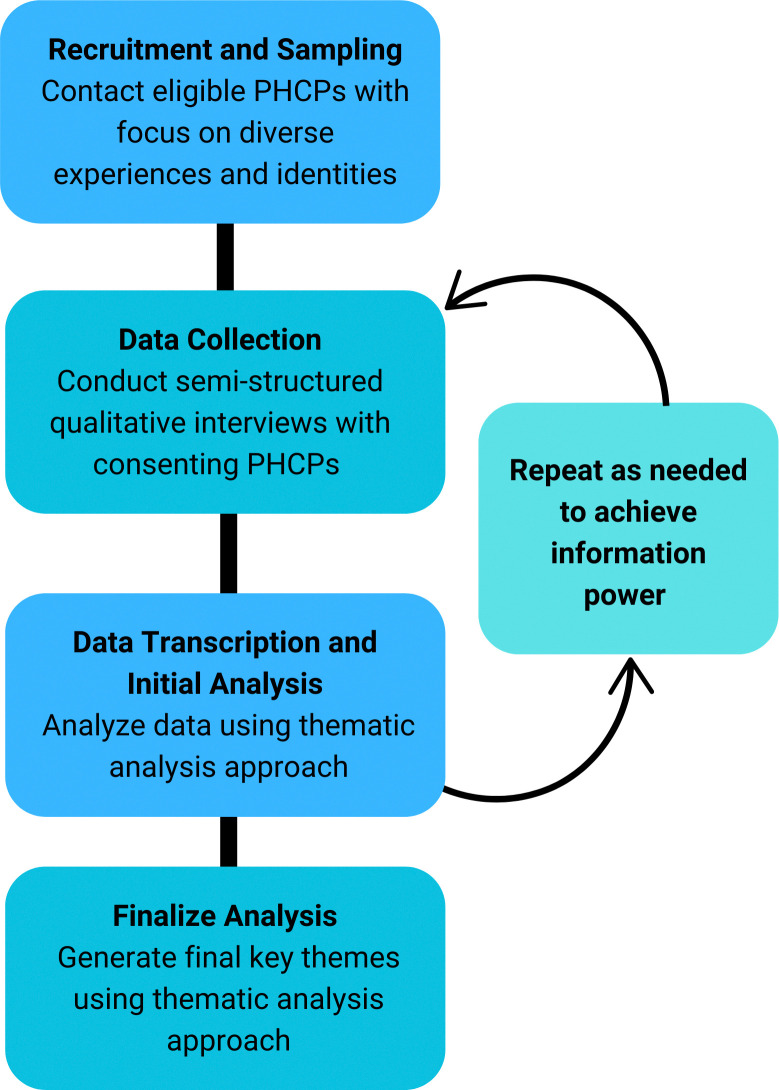
Workflow of study-related procedures. This diagram depicts the workflow of study-related procedures, including recruitment, sampling, data collection, transcription and analysis.

This qualitative inquiry is grounded in the COM-B framework and uses semi-structured interviews and thematic analysis to capture the perspectives of PHCPs practicing in Nova Scotia. Ethical approval was granted by the Nova Scotia Health Research Ethics Board (File #1027028). Fig 1 depicts the workflow of study-related procedures.

### Participants

Individuals were eligible to participate if they were practicing as PHCPs in Nova Scotia at the time of recruitment. Individuals were not eligible to participate if they were retired PHCPs, were practicing in a field other than primary health, or were not practicing in Nova Scotia. PHCPs were recruited through local contact and by reaching out to those who expressed interest in further research participation on a previous quality improvement questionnaire developed by the Division of Digestive Care and Endoscopy. In total, we approached 20 prospective participants. Five were unable to commit enough time for an interview. Purposeful sampling from all provincial health zones and in academic and private practices occurred. Participants were recruited with the goal of having diverse representation by age, gender, years in practice and practice setting (i.e. solo, group and collaborative practices). There was no minimum or maximum for participant age or years in practice, as long as individuals met the inclusion criteria. Participant recruitment and data collection occurred simultaneously. Sample size was determined based on information power. Information power refers to the number of participants needed to reach ‘power’ in qualitative research [20]. Using the information power model, the sample size is assessed based on the study aims, sample specificity, established theory, quality of interview dialogue, and analytic approach [20]. The number of participants that will be interviewed may change based on information power. An interim analysis was performed after 10 interviews to assess for information power. It appeared that we were nearing information power, so an additional 5 interviews were completed. All participants provided written consent using Nova Scotia Health’s e-consent process, then contacted by the research associate to complete a questionnaire containing key demographic and practice related questions and to schedule a virtual interview.

### Data collection

One-on-one semi-structured interviews with PHCPs were conducted by two interviewers with participants between September 1, 2021 and May 1, 2022. The interviewers were both women and had experience conducting qualitative research in the field of gastroenterology. The interview guide contained a series of 31 closed and open-ended questions and 8 sociodemographic questions to permit for exploration of rich qualitative themes and was developed through the collaboration of our interdisciplinary team, including gastroenterologists, family physicians, patient research partners and administrators. The COM-B behaviour change wheel domains (i.e. Capability, Opportunity, and Motivation) were used to guide question development in the interview guide to ensure implementation barriers were addressed comprehensively. The semi-structured interviews were conducted virtually, and audio recorded by the research associate using a secure video conferencing platform. Consent for audio recording occurred before the interview at the time of consent. Each interview lasted approximately 45–60 minutes.

### Data analysis

Transcripts were auto generated with the use of transcription software and reviewed for quality control. All transcripts were anonymized using a research ID. Transcripts were uploaded into NVivo Pro (Version 12) for data management. Analysis followed Braun and Clark’s thematic analysis approach [21]. Transcripts were read and reread by a single coder to familiarize themselves with the data, and then coded. Initial or first round latent coding was used to organize the data and assess for data saturation. A second round of coding was employed to further organize codes into key candidate themes. Organizing data into themes was an iterative process and required multiple passes of the data and reconfiguring related concepts to generate the final key themes. Theme names were refined, defined, and named. Key themes from the data were then organized by the three main COM-B concepts when writing. An audit trail was maintained to enhance trustworthiness of data. Member checking did not occur given the busy schedules of PHCPs.

## Results

**Fig 2:**
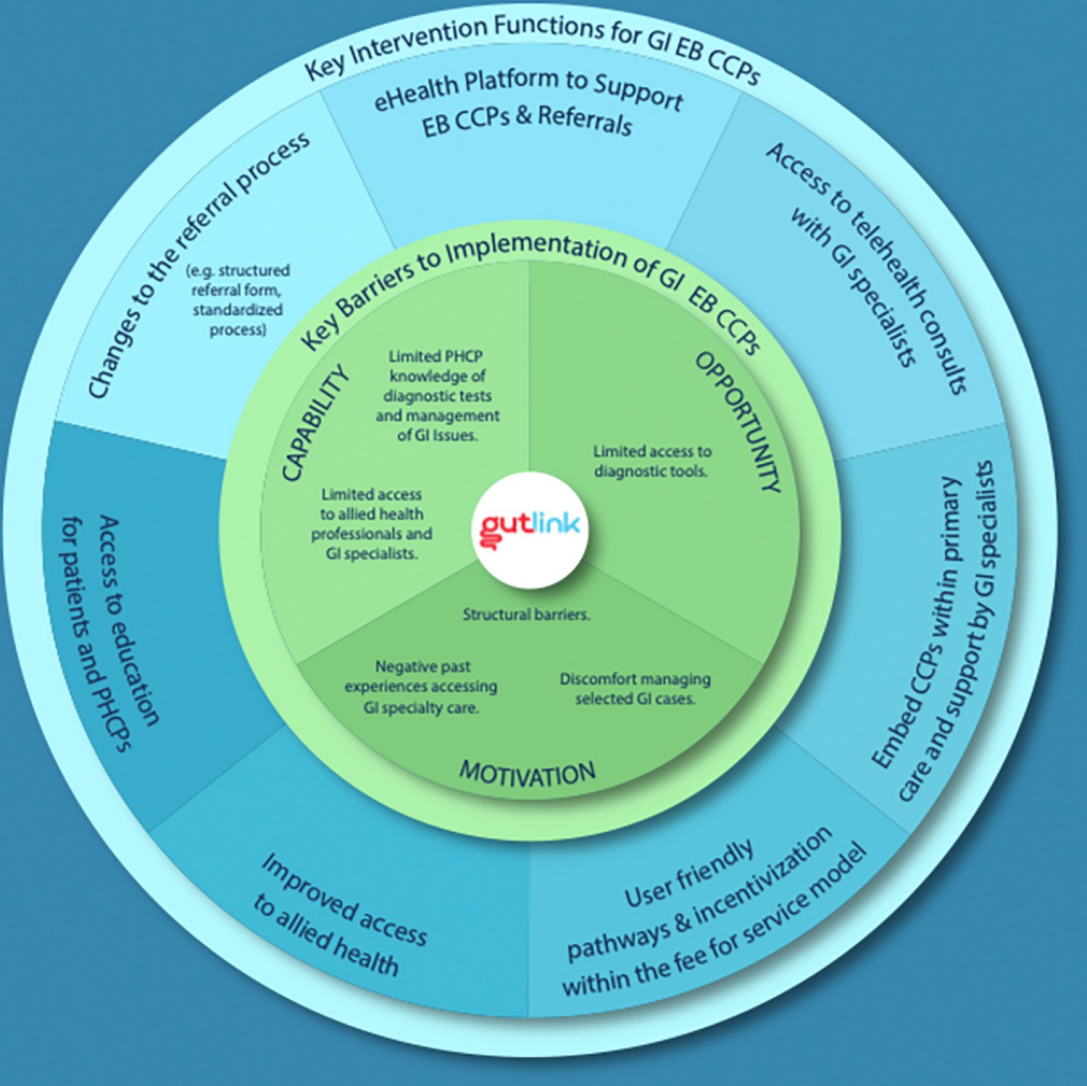
Key barriers to implementation of GUT LINK evidence-based care pathway. The green inner circle of the figure highlights key barriers to implementing an evidence-based gastrointestinal clinical care pathway, as reported by primary healthcare providers in Nova Scotia, Canada. The blue outer circle highlights potential interventions that could address said barriers.

A total of 15 PHCPs were interviewed. All PHCPs were physicians working in family practices in Nova Scotia. Participants were recruited across solo, group and collaborative practices. The mean age of participants was 42 years (SD = 10 years). The majority of participants identified as women (n = 11, 73%) and most practiced in urban areas of the province (n = 12, 80%). Additional sociodemographic data can be found in Table 1. Three key themes were generated and have been organized according to the domains of the COM-B implementation science framework: 1) Capability, 2) Opportunity, and 3) Motivation (Fig 2).

**Table 1:** Participant sociodemographic characteristics (N = 15).

Sociodemographic Marker	N (%)
**Gender**	
Man	4 (26.7)
Woman	11 (73.3)
**Ethnicity**	
White	11 (73.3)
South Asian	3 (20)
Arab	1 (6.7)
**County of Practice**	
Colchester	3 (20)
Dartmouth	1 (6.7)
Halifax	9 (60)
Kings	1 (6.7)
West Hants	1 (6.7)
**Health Zone**	
Central	13 (86.7)
North	1 (6.7)
West	1 (6.7)
**Urban/Rural Practice**	
Urban	12 (80)
Rural	3 (20)
**Practice Structure**	
Solo Practice	2 (13.3)
Group Practice	5 (33.3)
Collaborative Practice	6 (40)
Other	2 (13.3)

Self-reported sociodemographic characteristics from participating primary healthcare providers (N = 15)

### Theme 1: PHCP knowledge and access to allied health (Capability)

Most PHCP-defined barriers were linked to their perceived lack of knowledge of digestive diseases. Some PHCPs reported having a good knowledge of managing symptoms and understanding types of medications and lifestyle interventions. Yet, most reported limitations in GI knowledge as they were required to maintain a breadth of knowledge on all health conditions:

*“I feel like I have my family medicine work up, but then I worry that maybe there’s something newer that I don’t know about… I finished residency, you know, five years ago, but things change very quickly… And so, I’m always a little worried that I’m missing out on, you know, a new recommendation that my patients aren’t getting without that kind of feedback or expertise.”*

Several PHCPs reported not having knowledge of different diagnostic tests. Although some were aware of ROME, a diagnostic tool for IBS, few had applied the guidelines in their practice. PHCPs cited not having the tool readily available and having access to outdated materials as being key barriers to its application. Secondly, several PHCPs described not having knowledge about tests available to them or when they should order them:

“[*it is] Really embarrassing, I didn’t even know the first four tests were things that existed that one could order.”*

Even when they did order tests, some PHCPs were unsure of how to interpret the results.

*“Yeah, because we don’t have any education around what those tests mean: your stool pH, your electrolytes, your stool eosinophils, elastase…So, ordering tests that we’re not quite sure how to interpret isn’t very useful or safe.”*

PHCPs indicated that they wanted continued education on GI management and, particularly, about testing and interpretation.

“*I think we need a little bit more education to know what tests we should choose from because these are all very expensive tests, and they should be used with good judgment. So, I think the family doctors need to be coached or taught a little bit more about what other tests to use…”.*

PHCPs also cited limited access to allied health professionals, including GI specialists as being a barrier to care. Many indicated that they were not satisfied with access to GI speciality care:

*“Right now, I guess I’m not satisfied, I feel badly saying that. It’s not because people aren’t working hard, and are busy but I just I find, I worry for my patients because I see all the folks that are two to three years overdue for their routine screens, and then there’s the new people that I’m trying to get seen. So, yeah, I guess I hate to say it, but I am dissatisfied.”*

They reported long wait times and limited communication with GI specialists, which add to PHCPs’ already busy caseload. As a default, many PHCPs reported referring patients to general surgery.

“*I know that I can’t get access to [our GI specialist] so I send a lot of stuff I normally would to our general surgeons.”*

### Theme 2: Access to diagnostic tools (Opportunity)

PHCPs reported limited access to diagnostic tools as being a key barrier to accessing care, potentially impacting the ‘opportunity’ to adopt the intervention. Some PHCPs indicated they experienced challenges accessing the correct tests for their patients, including limited access to fecal calprotectin tests. Some tests could only be requested by a specialist which prevented access given limited communication and access to speciality care:

“*The stool calprotection–I have ordered in the past but that’s always been at the direction of a gastroenterologist. My understanding is family doctors can’t order that in central zone.”*

PHCPs also reported difficulties accessing endoscopy due to their inability to perform the test. This is problematic as endoscopy is required for diagnosis since some digestive diseases require endoscopic evaluation to exclude alternate diagnoses:

*“Well, I think the thing is I know how to take the work up to a certain point, but my understanding is often those are diagnoses of exclusion and part of the exclusion criteria, often involves scoping to ensure there is nothing else going on… but I can’t scope.”*

### Theme 3: Comfort with managing cases and optimism (Motivation)

Most PHCPs indicated they felt comfortable managing digestive disease within the family practice, and believed it was part of their role as a PHCP. Yet, some PHCPs highlighted instances where they did not feel comfortable managing cases, such as patients with complex conditions, including old age, and multiple comorbidities. PHCPs also described patients who wanted confirmation of their diagnosis from a specialist and not management from their PHCP:

*“So very often, it is the patient who is not convinced with the diagnosis and want to get a more definite opinion from a specialist, so I have to refer them…, to reassure their mind that this is a [correct] diagnosis that I have made.”*

Some discomfort was also attributed to uncertainty about referrals, such as not knowing how long a patient may wait to be seen or which specialist had the shortest wait list. Finally, some concerns were linked to the busy nature of family practices. Some PHCPs were concerned that they did not have enough time to properly manage complex cases:

*“I just feel sort of overwhelmed at my own family practice. I just don’t think I have time to, I don’t, I don’t feel I have time to manage my own patients my own practice is well oversubscribed. I suppose that’s the other thing with functional disorders too, is that they’re very time consuming.”*

A few PHCPs also perceived a need to determine the extent of their responsibility to manage care, as some aspects of case management may align better with specialist management. Many PHCPs indicated that extra support from GI specialists would support their ability to manage GI issues within their practice:

*“… if I have done some basic tests and I’m not able to come to a conclusion… I will require the gastroenterologist to help me, confirming the diagnosis. And for the management, obviously once a diagnosis is clear what it is, the management depends on who would be the right person to treat that condition.”*

Many PHCPs noted a lack of optimism given their past experiences trying to access GI care. Some of this lack of optimism was attributed to historically long wait times for speciality care:

*“…weekly I have issues that I think are appropriate for gastroenterology but given their waitlist and the number of referrals they get declined, I refer less and less to them, knowing that my patients won’t get seen or its most likely to be rejected.”*

When patients had to wait for specialty care, they often became anxious and PHCPs had to “tide over” patients by ordering other tests or trying to diagnose within the family practice. One PHCP shared:

*“Absolutely I [am] managing things that are not within my scope of practice. I’m tiding patients over; I’m watching patients get sicker. All while waiting for necessary testing and investigations. And that’s very frustrating for both me and the patients and their families.”*

One PHCP indicated that clinical care guidelines could help with case management while waiting for specialist care. The long wait times left many PHCPs feeling anxious. They reported wanting reassurance from a GI specialist that nothing was missed in their diagnosis:

“*And can you please confirm or make sure that I’m not missing anything else. I’m fairly confident this is what it is, but the patient is demanding to see you for any further optimization.”*

In two cases, PHCPs reported patients dying while waiting for access.

Some PHCPs also reported feeling pessimistic considering limited support from the broader healthcare system. Some PHCPs understood that the wait times for specialty care were beyond the control of specialists and a result of chronic underfunding and understaffing of GI care in the province:

*“I believe that they are severely understaffed underfunded. There are unfortunately politics and rules around how patients can be booked and how many scopes a day can happen you know, based on hospital policy union mandates. So, I realize it’s an entirely complex, there’s not one person to blame. And it’s not because our specialist colleagues don’t want to work and in fact, I would argue, they’re all working very hard. There’s just not enough of them and they’re not being used efficiently.”*

Nevertheless, some also felt under supported by specialists who did not seem to understand the clinical realities of primary care:

*“So, that instead of being like, “Oh, here’s another one the family doctor missed” when things come [a specialist’s] way [they] can realize a huge number of things we’re already dealing with, with the complete lack of resources. I can barely get my patients’ bloodwork and X-rays…”*

## Discussion

Findings from this study demonstrate that PHCPs experience numerous behavioural and environmental barriers related to accessing speciality GI care and implementing GI evidence-based pathways in real-world clinical practice. Although few facilitators were identified by PHCPs, those that were identified may provide insight into what is currently working well in the healthcare system and would support immediate implementation of a clinical care pathway. A few PHCPs indicated that they have a good existing knowledge of how to manage GI symptoms. This existing knowledge suggests that some PHCPs likely already have a strong understanding of existing clinical guidelines, but would benefit from the extra support provided by the pathway to link patients to specialty care. Additionally, this knowledge of what is already ‘working’ can help guide resource allocation during pathway development and implementation to support other ‘weaker’ areas for optimal uptake.

Barriers were primarily highlighted by PHCPs. Some of the PHCP-perceived barriers have previously been identified in the medical literature and, thus, may not be surprising. For example, long wait times and confusing referral systems have previously been identified by both PHCPs and patients as being barriers to care [9, 10]. A previous survey of family physicians in Nova Scotia on access to IBD specialty care also identified poor communication with GI specialists and the feeling of being unsupported as key barriers [9]. This current research focused specifically on understanding the implementation of evidence-based GI care in the real-world clinical practice of PHCPs. New themes emerged from this study. For instance, some family physicians identified having limited access to and/or being unable to interpret certain evidence-based pathway components such as diagnostic tests (i.e., fecal calprotectin); to the best of our understanding, this has not previously been noted in the literature.

Previous research has also observed variation in the uptake of evidence-based clinical GI guidelines, despite the economic and health benefits of standardized care for digestive disorders [22, 23]. Some variation may be explained by the challenges posed by some of the barriers identified in our study, such as PHCPs’ limited time and knowledge [24]. Our use of the COM-B model (an evidence-based determinant implementation science framework) to develop the interview guide is a strength of this research, as it facilitates insight into how some key barriers may be addressed to support the development and uptake of a novel clinical care pathways in practice. We anticipate that implementing an evidence-based clinical care pathway, like GUTLINK, could address most, if not all, of the barriers identified by PHCPs. However, a roadmap relating to how to implement a clinical care pathway is lacking in the medical literature. Our research contributes to this gap in knowledge by identifying some key barriers to implementation which may inform other clinicians and decision makers wanting to implement their own GI clinical care pathway. We also acknowledge that additional study may be warranted to evaluate pathway implementation and identify best practices.

Key *capability* barriers centered on knowledge gaps and speciality care gaps. Co-designing the GUTLINK pathway with PHCPs would ensure that these knowledge gaps are accounted for. The GUTLINK evidence-based pathway implementation strategy should also include an educational component for PHCPs that is responsive to their clinical needs or guide decision making with clinical decision support to alleviate concerns about missing a diagnosis and to build confidence in their knowledge that they are providing the most current evidence-based care. Implementation of virtual decision-making tools in IBD care have been shown to improve quality of care and patient outcomes. In Australia, a tablet-based decision-support tool was evaluated for ulcerative colitis patients. One cohort attended a clinical follow up where they discussed 13 process indicators without use of the decision support tool. The second cohort used the decision support tool with their clinician prior to consultation to generate a suggested management plan that could be discussed. The cohort that used the decision support tool showed improvement in psychological well-being, preventive care, and disease activity management [25]. GUTLINK implementation strategies should also support environmental restructuring that allows for improved virtual access to evidence-based specialty care and allied health professionals through a virtual medical neighborhood. Previously established virtual health homes, such as Project ECHO (Extension for Community Health Outcomes), have demonstrated improved clinician collaboration and access using virtual conferencing [26]. Project ECHO is a virtual consultation tool that remote clinicians can use to consult with gastroenterologists at academic teaching hospitals. The speciality consults provide expert advice on clinical cases, support clinical education and development of care pathways [26].

PHCPs cited poor access to diagnostic testing as being a key *opportunity* barrier. It is difficult to fully address this barrier through a virtual pathway since some of the key issues link to PHCPs’ scope of practice and inability to perform scoping tests. However, access to diagnostics could be improved by enabling pathway-mediated facilitation of diagnostic testing that PHCPs currently lack access to, as well as improved communication with specialists. A PHCP and patient-facing educational component may also reduce the need for diagnostic testing by reassuring both PHCPs and patients that testing is not required based on their presenting symptoms. Many PHCP-identified barriers were *motivational in nature*, suggesting that training and enabling PHCPs to use GUTLINK may support consistent uptake. For instance, providing regular real time engagement and training support may help PHCPs integrate GUTLINK into their practice. Persistent engagement and collaboration with PHCPs and other health professionals through the pathway may also demonstrate time efficiency and commitment to using the pathway. Ensuring that the pathway provides improved access to necessary diagnostics testing, clinical decision support, greater referral efficiencies, and improved communication with specialists may also have tangible workflow benefits for PHCPs in their practices. Ongoing engagement to adjust the pathway as needed and emphasis of shared clinical goals may exemplify support and respect for the PHCPs’ role in patient management.

### Limitations

There are a few limitations to this research. First, a small sample size may mean that not all physician experiences were captured in this study. PHCP recruitment into research studies is notably difficult, especially in qualitative research which often requires a greater time commitment. Secondly, this research was conducted in the context of a publicly funded primary healthcare system that uses a fee-for-service model. We recognize that other funding and compensation models exist and may create different barriers to care. Nevertheless, we believe our findings are still important lessons for clinicians who are contemplating the implementation of a clinical care pathway in a resource limited environment. Future research could examine implementation of evidence-based GI clinical care pathways in other systems, as well as PHCP-identified solutions to addressing barriers to pathway implementation. Additional research on facilitators to pathway implementation, as well as additional information about the potential impact of PHCPs’ clinical volume, proximity to GI specialists, and utilization of other evidence-based resources to guide clinical decision making would also be beneficial.

## Conclusions

Despite playing an important role in the management and care of patients, PHCPs encounter several barriers to accessing speciality GI care and implementing an evidence-based pathway for specialty GI care, spanning personal knowledge of GI issues to structural barriers linked to funding and support. Although a clinical care pathway may support improved quality of patient care, clinical guidelines are rarely consistently implemented in primary healthcare. Our research elucidates these barriers and suggests potential ways that these barriers could be addressed to develop and ensure consistent uptake of a novel GI clinical care pathway. Although some barriers can be addressed through a clinical care pathway, we acknowledge that structural barriers, such as limited funding for multidisciplinary GI care, must also be addressed at an institutional and governmental level to adequately address population need for digestive health disorders, to improve the likelihood of successful evidence-based care pathway implementation, and to improve access to high quality care.

## Supporting information

S1 File Interview guide. Interview guide featuring questions and probes that were posed to participants.
(PDF)

## Abbreviations

GI: gastrointestinal
IBD: inflammatory bowel disease
IBS: inflammatory bowel syndrome
NS: Nova Scotia
PHCP: primary health care provider
