# Supplementary material for: Primary healthcare provider-perceived barriers to implementing an evidence-based pathway for undifferentiated lower gastrointestinal tract symptoms: A qualitative inquiry
Source: PLoS One. 2024 Dec 31;19(12):e0313201. doi: 10.1371/journal.pone.0313201 (PMC11687702; doi:10.1371/journal.pone.0313201)
Supplement: S1 File — Interview guide featuring questions and probes that were posed to participants. (PDF) [file pone.0313201.s001.pdf]

1.2 Preamble for semi-structured PHCP interview:

First of all, the Division of Digestive Care and Endoscopy would like to thank you for taking the time out of your busy schedule for engaging with us today. Although this does not come close to compensating you for your time, we would like to provide a digital gift card to Chapters as a small token of appreciation for your time.

This interview is intended as a first step toward better understanding how we can improve access to high quality digestive care for patients suffering with gastrointestinal disorders here in Nova Scotia, a region that is under resourced in both primary health care and specialty digestive care.

This topic of this interview will seek to better understand the barriers and facilitators to providing care for patients with undifferentiated lower gastrointestinal tract (U-LGIT) symptoms in your clinical practice. Symptoms may include diarrhea, constipation, bloating, or pain with or without rectal bleeding. Such symptoms may be indicative of a range of gastrointestinal diagnoses including, but limited to, celiac disease, irritable bowel syndrome (IBS), inflammatory bowel disease (IBD), or colorectal cancer (CRC).

The results from this interview will be critical to inform and tailor the design of integrative strategies that will improve access to specialist facilitated or directed digestive care for Nova Scotians. This can only be achieved through integration and close collaboration between specialists and primary healthcare providers.

We anticipate this interview to take approximately 45 Minutes. If at any time you do not feel comfortable answering a question, or you do not understand a question, please let us know. The answers to these questions will be de-identified. The session will be recorded in order to facilitate qualitative data analysis.

1.3 Virtual PHCP interview script

Area	Question Stem	Probes
**Experiences & Perceptions**	How often do you refer your patients to a gastroenterologist (for all diagnoses)?	How many times per clinic? per week?<br><br>What proportion of those referrals do you feel require the involvement of a specialist to provide a diagnosis or treatment?
	What is the most common reason you refer your patients to a gastroenterologist?	
	Would you consider lower GI complaints to be common in your clinical practice?	

	How often do you encounter lower GI complaints in your family practice (i.e. constipation, abdominal pain, diarrhea)?	How often would it be the presenting problem? A secondary problem?<br><br>How many patients per week? Per month?
	How comfortable are you with the work-up of U-LGIT symptoms?	What components, specifically, are you uncomfortable with?
	How would you describe your knowledge around the work-up and diagnosis of U-LGIT symptoms?	(i.e. poor, fair, good, very good)?
	Functional disorders are characterized by GI symptoms that are not attributable to a structural or biochemical casue and are thought to represent dysfunction of gut–brain interaction. The most common example is Irritable Bowel Syndrome (IBS).<br><br>How comfortable are you with being able to distinguish functional lower GI disorders from those caused by organic disease?	What specific aspects are the most difficult for you to manage?
	Are you aware of any approaches to diagnosing IBS or investigating patients with U-LGIT symptoms?	Do you use a guideline?<br><br>Have you heard of the ROME classification? Do you use it?
	Do you use any specific resources to guide you in the diagnosis and management of U-LGIT? If so, which resources?	i.e. apps, UpToDate, printed material, google search, journal articles, published guidelines, etc. ?
	What do you think are the most important things to assess when a patient presents with U-LGIT symptoms?	e.g. symptoms (no BM's a day, stool consistency, abdominal pain, rectal bleeding)<br><br>medication review,<br><br>blood work<br><br>imaging tests<br><br>rectal examination, abdominal examination
	When considering your typical workup for U-LGIT symptoms:<br>- Do you order lab tests? Which one(s)?	

<table>
<tr><td rowspan="3"></td><td>- Do you imaging tests? Which one(s)?</td><td></td></tr>
<tr><td>[ZOOM POLL 1]

Q1. Please look at this list of specialized lab tests.

- Stool pH
- Stool electrolytes
- Stool eosinophils
- Stool elastase
- Stool lactoferrin/calprotectin
- Tumor markers (e.g CEA, CA 19-9)

Do you ever use specialized lab-based diagnostic tests? If so, in what situation?

Q2. Please look at this list of specialized lab tests.

- Stool pH
- Stool electrolytes
- Stool eosinophils
- Stool elastase
- Stool lactoferrin/calprotectin
- Tumor markers (e.g CEA, CA 19-9)

If you had better access, do you see any of these playing a role in your clinical practice?</td><td></td></tr>
<tr><td colspan="2">[ZOOM POLL 2]

Not all patients presenting to primary care with lower GI symptoms are referred to a specialist. We would like understand what patient characteristics, symptoms, and test results would prompt you to refer a patient to a gastroenterologist.

Considering a typical patient presenting with U-LGIT symptoms (e.g. loose BMs, constipation, abdominal pain, bloating), for each of the following please indicate if it would prompt you to refer the patient to a gastroenterologist.

[Response options to each item will be Refer either or Do not refer]</td></tr>
</table>

<table>
<tr><td rowspan="4"></td><td colspan="2">

- Abdominal pain or cramping related to BMs
- 5 BM’s a day
- Watery stool consistency
- Nocturnal bowel movements
- Constipation with a sensation of fullness, blockage, or incomplete emptying
- Pencil thin stools
- Intermittent blood on toilet paper with wiping
- Hard stools or difficulty passing stools (straining)
- Hemorrhoids on rectal examination
- Bloody diarrhea
- Unintended weight loss
- Symptom onset after > age 50
- 1st degree family history of CRC or IBD
- 2nd degree family history of CRC or IBD
- Anemia
- B12 deficiency
- Iron deficiency or iron deficiency anemia
- Elevated CRP
- Elevated fecal calprotectin

</td></tr>
<tr><td>**[ZOOM POLL 3]**

Q1. How comfortable are you with the management of lower GI disorders overall?

Q2. How comfortable are you with the management functional lower GI tract disorders specifically?</td><td>Responses will be on a scale of 1 – 10 (1 not at all comfortable, 10 very comfortable)

Responses will be on a scale of 1 – 10 (1 not at all comfortable, 10 very comfortable)</td></tr>
<tr><td>When you suspect a functional lower GI disorder (e.g. IBS), what proportion of patients do you refer to GI?</td><td>What is the main reason that you refer patients with suspected functional lower GI disorders?</td></tr>
<tr><td>When you suspect a functional lower GI disorder (e.g. IBS), do you worry about missing serious organic disease?</td><td>What is the main concern?
(i.e. medicolegal vs. discomfort with possibility of missing disease with all screening tools, lack of experience/knowledge</td></tr>
</table>

	What assistance are you seeking when you refer a patient with a suspected functional lower GI disorder to a gastroenterologist ?	Medication recommendations?<br>Endoscopy necessary to diagnose IBS?<br>Reassurance for patient?<br>Reassurance for yourself?<br>Other
	When you do require a specialist for assessment of U-LGIT symptoms, how satisfied are you with access to these services?	
	Tell me about your experiences with accessing GI care for your patients?	Have your experiences been generally positive? Negative?<br>*Note: if negative response, ask "how did that make you *feel?" and*<br><br>*Has this experience affected your referral patterns (likelihood of referral, patterns of referral, how specialty care is accessed (phone call, paper based referral)?*
	Does access to specialist services (or lack of) have an impact on your clinical practice?	Examples: number of clinical visits with patients? Phone calls to patients?, additional tests?, phone calls to specialists/on-call services? re-referrals and additional letters?, calls to specialist offices?)
	**[ZOOM POLL 4]**<br>Q1. How would you rate the current process for a referral to a gastroenterologist for U-LGIT symptoms?<br><br>Q2. Compared to accessing other specialty care, where would the current process of accessing gastroenterologist care rate?	Responses will be on a scale of 1 – 10 (1 terrible, 10 excellent)<br><br>Worse<br>About the same<br>Better
	How important do you think you think it is for a primary care clinician to be able to diagnose and manage U-LGIT symptoms? To differentiate functional GI conditions (IBS) from organic disease (IBD) ?	Why or why not?

	What do you think the role of a primary care clinician is in the diagnosis and management of lower GI disorders? Is it different for irritable bowel syndrome (IBS) vs inflammatory bowel disease (IBD)?	Why or why not?
	What do you think the role of a gastroenterologist is in the diagnosis and management of lower GI disorders? Is it different for IBS? IBD?	Why?
**Barriers & Facilitators**	Do you have access to all of the diagnostic test/interventions that you need to investigate U-LGIT symptoms?	If not, how could access be improved? What other tests or investigations would you like to have access to?
	What does the access to diagnostic testing and procedures for your work-up of U-LGIT symptoms look like in your practice?	
	If you had access to clinical criteria for diagnosing lower GI conditions (e.g. ROME IV), would it be helpful?	
	If you had access to fecal calprotectin would it help you in clinical practice?	
	What does your access to important allied health professionals (i.e. pharmacist, dietician, NP, RN, LPN, psychologist, nutritionist, social worker, etc) look like?	How often do patients make use see a pharmacist? dietician? NP? RN or LPN? health psychologist? Social worker?<br><br>What proportion of patients who could make use of allied health providers actually see them?<br><br>How could things be optimized in this regard?
	Do you access other resources for assessment of patients with U-LGIT symptoms (i.e. refer to the emergency department, surgeon, internist, specialists in another health zone)?	If so, what is your experience with referrals to these services?
	What resources (infrastructure and human), processes, or information would help you to implement a Clinical Care Pathway for IBS?	

	What would optimal access to a gastroenterologist look like for you?	When answering this question consider the relative under resourcing of both Primary and specialty care services and comment on the referral and triage process, method of communication<br><br>Are there things you would like to change in existing process for referral, triage and communication with GI specialists?<br><br>-Referral: unformatted letter? Structured form? Web-based tool for determining eligibility for GI referral?<br><br>-Triage process: communications re: required investigations, requests for additional information, patient waitlisting for clinic/direct access endoscopy?<br><br>- What do you think the process for communication between specialists and PHCPs should look like for case management of LGIT symptoms?
**Intervention Functions & Implementation Strategy**	How open would you be to access a specialist-facilitated* collaborative care pathway for U-LGIT disorders?	*a pathway whose components could be facilitate by specialists (for example, if access to GI specific dietary interventions are lacking in primary healthcare, the specialist center can facilitate access to these)
	What do you believe would be the value in having an evidence-based, specialist-facilitated, collaborative care pathway for the investigation and management of U-LGIT symptoms?	
	**SHARE SCREEN: CRUDE PATHWAY**<br>The investigation and management of U-LGIT symptoms often requires access to tests and allied healthcare professionals.<br><br>Take a look at the evidence-based components involved in the diagnosis and management of U-LGIT symptoms:<br><br>- Symptom assessment<br>- Medical and family history review	"I AM GOING TO SHOW YOU A SAMPLE PATHWAY"<br><br>a. What components *would* be achievable or accessible in your practice?<br>b. What components *would not* be achievable or accessible in your practice?

	- Medication review<br>- Physical exam<br>- Investigations (appropriate labs, stool tests, imaging tests, endoscopy)<br>- Results communication / patient reassurance and counselling<br>- Dietary interventions<br>- Lifestyle interventions<br>- Psychological counselling<br>- Medication trials	-Why or why not?<br>d. Would it be beneficial to have access to the components you are lacking?<br>e. *What would be the ideal method to access these services?*<br>*f. Is it feasible to implement this type of approach to the diagnosis and work up of* U-LGIT *symptoms in your practice?*<br>*g. If yes, what infrastructure and process does your practice have that could facilitate using a pathway like this?*
	Would a specialist-facilitated, collaborative care pathway for lower GI conditions be useful for your current clinical practice?	What would motivate you to use such a pathway? How would this be helpful?
	Would there be any benefit of having a specialist-facilitated, collaborative care pathway integrated into a GI referral process?	
	How do you currently incorporate clinical care pathways in you practice?	
-	What do you feel is the best way to implement care pathways in your clinic ?	
	In your practice, who do you see implementing a care pathway (MD, LPN, RN, NP, dietician etc.)?	
	Does your practice currently use an electronic medical record? If so, please describe your current EMR.	**If your practice does not use an electronic medical record, please briefly describe the barriers to an EMR.
	How open are you to using virtual platforms/telehealth? e-health technology?	In general? Specifically for the purposes of referral? For accessing clinical care pathways and decision support tools?
	How comfortable are you with using e-health technologies?	Did you start using any new technology due to the COVID-19 pandemic?<br>Has the COVID-19 pandemic changed your comfort level? How so?

	Do you foresee any challenges regarding the use of a virtual/telehealth platform in your practice?	- In general? To support facilitation of referrals? To access specialist-facilitated, collaborative care pathways?
	Would you be open to using e-health/virtual platforms in order to participate in consultations with specialists?	If not, why might this be (i.e. inadequate time during the work-day, comfort level, not within “scope of practice”, not remunerated, etc)?
	What do you think the optimal implementation of care pathways for LGIT symptoms would look like your clinical practice?	How could GI specialists best support this?
	Would you be open to using e-health/virtual platforms in order to participate in educational initiatives?	If so, what type of educational initiatives would be of greatest value to your practice? (i.e., case based reviews, evidence based topic reviews)
	What would you see as the optimal way to incorporate education relating to the diagnosis and management of LGIT symptoms?	For PHCPs ?<br>For patients ?<br>For families ?
	How interested would you be in engaging with a gastroenterologist to develop an evidence-based care pathway for the diagnosis and management of lower GI disorders if it led to improved and streamlined access to diagnostic testing, treatments and, when appropriate, specialist consultation?	
	What resources do you believe would be the most helpful for you when it comes to supporting the assessment and management of patients with lower GI conditions?	
	What is the best means of delivery of these resources (i.e. telephone, email, traditional website, interactive website with decision trees, pre-recorded educational videos, live educational sessions (virtual or in-person), etc.)?	
